# Structure of Novel Phosphonium-Based Ionic Liquids with S and O Substitutions from Experiments and a Mixed Quantum-Classical Approach

**DOI:** 10.1021/acs.jpcb.5c00129

**Published:** 2025-03-27

**Authors:** Raphael Ogbodo, Gobin Raj Acharya, Ho Martin Yuen, Nicole Zmich, Furong Wang, Hideaki Shirota, Sharon I. Lall-Ramnarine, James F. Wishart, Andrew J. Nieuwkoop, Claudio J. Margulis

**Affiliations:** † Department of Chemistry, The University of Iowa, Iowa City, Iowa 52242, United States; ‡ Department of Chemistry and Chemical Biology, Rutgers University, Piscataway, New Jersey 08854, United States; § Department of Chemistry, Queensborough Community College-CUNY, Bayside, New York 11364, United States; ∥ Chemistry Division, Brookhaven National Laboratory, Upton, New York 11973-5000, United States; ⊥ Department of Chemistry, Chiba University, Chiba 263-8522, Japan

## Abstract

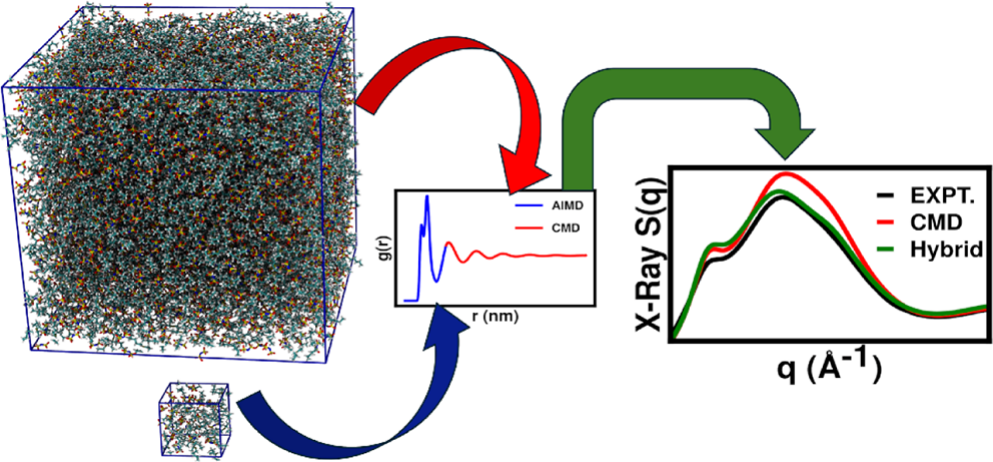

This article presents experimental characterization information and synchrotron X-ray scattering measurements on a set of novel O- and S-substituted phosphonium-based ionic liquids (ILs) all coupled with the bis(fluorosulfonyl)imide (FSI^-^) anion. The ILs include the ethoxyethyltriethylphosphonium (P_**222(2O2)**_^**+**^) and triethyl[2-(ethylthio)ethyl]phosphonium (P_**222(2S2)**_^**+**^) cations, and we contrast results on these with those for unsubstituted triethylpentylphosphonium (P_**2225**_^**+**^). The article also introduces a physics-based protocol that combines classical force field studies on larger simulation boxes with classical and first-principles studies on smaller boxes. The method produces significantly improved *S*(*q*) functions in the regime which in prior publications we have associated with inter- and intraionic adjacency correlations. By understanding which shorter-range structural changes improve *S*(*q*) in the *q*-regime of interest, we are also able to pinpoint specific deficiencies in the classical force field model. The approach we take should be quite general and could help study other complex liquids on different length scales.

## 1 Introduction

**Figure 1:**
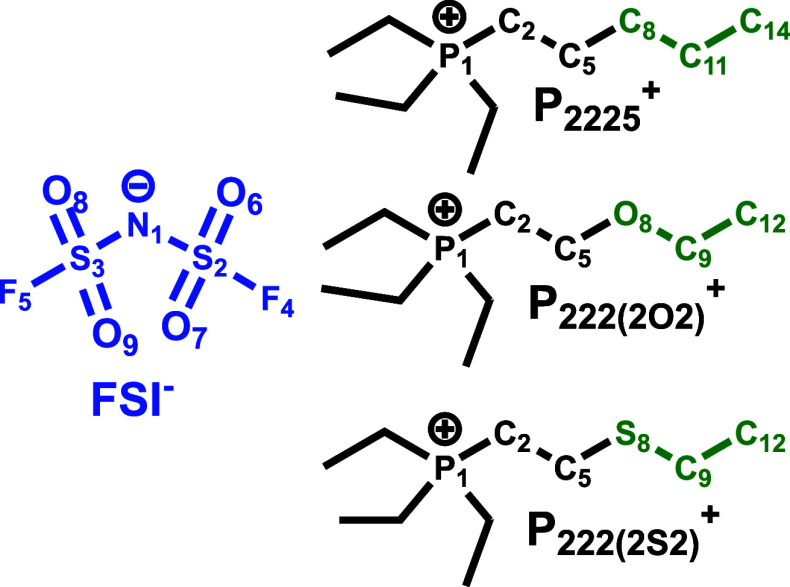
Ionic structures are color-coded to match the definitions for anion, cationic head, and tail used throughout the article. Atoms are labeled with numbers that are used in other figures to identify the corresponding species.

This article studies from an experimental and computational perspective a set of ILs developed in the Shirota lab^1^ depicted in Figure 1. Our original interest in phosphonium-based ILs stemmed from the advantageous properties that some of them appear to have, including reasonably low viscosities, appreciable conductivities, and low glass transition temperatures^2−7^ making them plausible candidates for energy applications.^7−9^ In addition, it is known that O-substitutions in cationic tails tend to result in ILs with even lower viscosities.^10−12^ Our understanding of thioether substitutions is less complete, but the Shirota group has recently published work on a few of these, studying their wettability and surface tension.^13^ Shirota and coworkers^1^ also characterized the spectral differences at low frequency (terahertz time-domain spectroscopy and Raman-induced Kerr effect) for the ILs depicted in Figure 1 and explained these in terms of the difference in strength of ionic interactions across systems (largest in P_**2225**_^**+**^/FSI^**–**^) and the distinct rates of vibrational dephasing across the group.

From multiple prior articles,^14−26^ we have learned that on the nanometer or longer length scales, the structure of ILs is fairly straightforward to predict. One will always find percolating charge networks in which positive and negative species are separated by a characteristic distance, and if the ILs include sufficiently long tails, there will also be other characteristic distances associated with the spacing across the strands in the percolating charge network. Most sensible physics-based models will capture these features and produce simulated structure functions, *S*(*q*), that in broad strokes can be described as reasonably correct. A more nuanced approach is required if one seeks to carefully examine correlations that are shorter in range. By this, we mean length scales nominally of ∼1 nm or less, which include most or all intramolecular correlations of the ions as well as the correlation between adjacent cations and anions. As it will become apparent from the results section, where we show experimental and computationally derived *S*(*q*) functions for these systems, this adjacency regime together with the charge alternation regime are the most important ones for the ILs shown in Figure 1 given that there are no significant tail domains that can produce a prepeak or first sharp diffraction peak.

If one chooses to stay within the realm of physics-based molecular dynamics (in which forces are computed from the gradient of a physical potential, as opposed to other successful methods such as machine learning), then the options for getting accurate structural information on the nanometer or subnanometer regime are not many. Classical simulations, even with polarizable force fields, will struggle with some of the shorter scale intra- and intermolecular details that require a true quantum chemical approach. On the other hand, ab initio molecular dynamics (AIMD) simulations can hardly sample at constant pressure the type of box sizes and time scales that give rise to properly converged *S*(*q*) functions even for ILs in the lower range of viscosity. In the methods section, we will describe a protocol we introduce in this paper where we attempt to obtain intermediate-range periodic liquid behavior from larger and longer classical MD (CMD) simulations, whereas the shorter inter- and intraionic behavior is well sampled from a collection of AIMD simulations starting from well time-separated classical MD frames. We will show that the shorter distance regime (*q* between 1 and 2.2 Å^–1^) is much better reproduced using this protocol.

## 2 Methods

The ILs used in this study were recently synthesized for their use in ref (1) and specific details associated with the synthesis of P_**222(2S2)**_^**+**^/FSI^**–**^ were reported in ref (6). P_**2225**_^**+**^/FSI^**–**^ and P_**222(2O2)**_^**+**^/FSI^**–**^ were synthesized as described in our prior work.^27^ The structure of the ILs was confirmed by ^1^H NMR and elemental analysis (<0.2%).^1^

### 2.1 Wide Angle X-Ray Scattering (WAXS) Experiments

The ILs were loaded in glass tubes with an outer diameter of 3 mm and flame-sealed immediately after being removed from the glovebox, which had an Ar atmosphere. The WAXS experiment was performed at beamline 11-ID-C from the Advanced Photon Source (APS), Argonne National Laboratory, with an X-ray beam size of 0.5 × 0.5 mm, a wavelength of 0.1173 Å, and energy of 105 keV. The sample-to-detector distance was 315 mm, calibrated with the standard reference material (Si SRM640c). The sample temperature was controlled with an Oxford Cryostream and equilibrated by waiting at least 5 min before data acquisition. Raw X-ray images were integrated using the program GSAS-II^28^ to determine the intensity in the aforementioned *q* range. The background was measured on an empty tube and subtracted from the raw data before further analysis. The total X-ray scattering function *S*(*q*) defined in eq 1 was obtained using the pdfgetX2^29^ code after performing background subtraction, as well as corrections for Compton, multiple, and diffuse scattering.
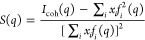
1

In eq 1, *i* labels atomic species, and *x_i_*, *f_i_*, are the corresponding fraction and X-ray form factor associated with species *i*.

### 2.2 Physical Characterization of Phosphonium Ionic Liquids (ILs)

Physical characterization was performed on ILs with water contents of less than 100 ppm, as measured by Karl Fischer titration.

**Viscosity:** Viscosities were measured with a Cambridge Applied Systems ViscoLab 4000 electromagnetic reciprocating piston viscometer housed in a moisture-controlled drybox fitted with a hygrometer. The temperature was regulated by a Lauda RM-6 circulating bath containing a 60/40 v/v propylene glycol/water mixture. The viscometer was calibrated with S6 and S60 viscosity reference standards from the Koehler Instrument Company. Measurements were taken when the moisture level was less than 1%. Viscosities were recorded at intervals between 0.1 and 95°C. The data were fit using the logarithmic form of the Vogel–Tammann–Fulcher relation,
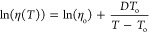
2and a value of 1 × 10^13^ cP was inserted at each sample’s glass transition onset temperature (*T*_g_) to anchor and stabilize the fitting so that different liquids could be more accurately compared.

**Conductivity:** Conductivities of samples were measured using a YSI 3200 m with a YSI 3253 dip-type conductivity probe with platinum black electrodes (modified to work as a fill-type electrode). The measurements were performed in a compressed-air-purged drybox when the moisture content was less than 1%.

**Thermal profile:** Glass transition onset temperatures of samples were obtained using a PerkinElmer Diamond differential scanning calorimeter and a liquid nitrogen cooling system with a scan rate of 5 °C/min over temperature ranges from −120 °C to 30 °C. Data were analyzed using PerkinElmer Pyris Data Analysis software.

**Density:** Densities were measured gravimetrically using calibrated 1 mL volumetric flasks. Measurements were performed in a moisture-controlled drybox when the moisture content was less than 1%.

### 2.3 Computational Methods

Our study involves classical and first-principles simulations used in combination to better understand liquid structure on different length scales. The sections below describe the protocols for classical and first-principles simulations, as well as how these are used in combination to produce an enhanced version of *S*(*q*). The version of *S*(*q*) that results from this combined approach matches experimental results significantly better in the *q*-region of interest than any of the individual CMD or AIMD results alone.

#### 2.3.1 Combined Simulation Protocol

We aim to produce *S*(*q*) at a combined MD/AIMD level of theory, taking advantage of less expensive larger-box classical sampling at long distances (properly capturing lower *q* phenomena) and first-principles ion conformational accuracy at shorter distances.

**Figure 2:**
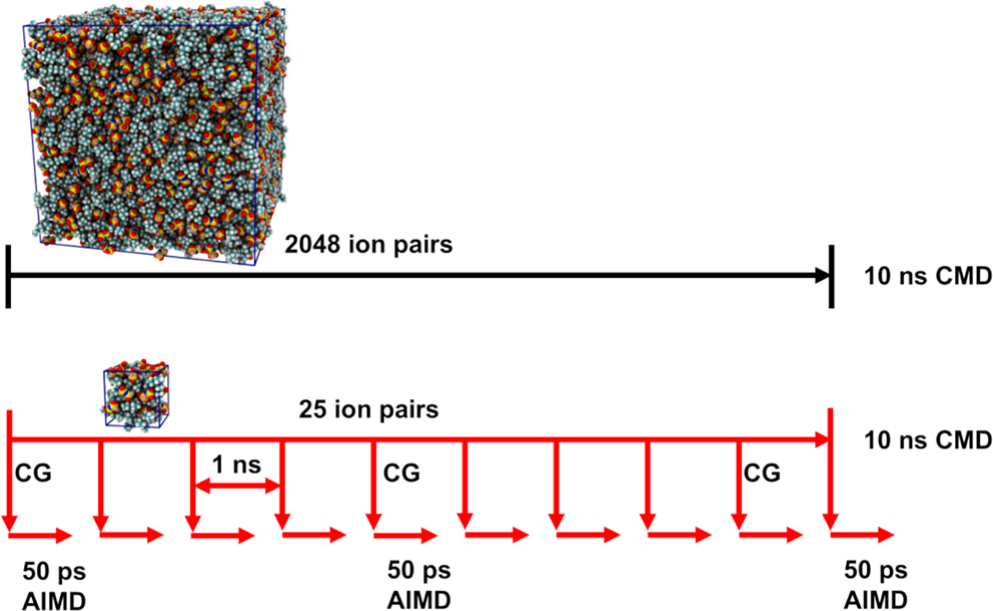
Graphical illustration of the production run simulation protocol.

Figure 2 pictorially describes the production run approach. As can be gleaned from the scheme, two types of classical simulations were concurrently run; one based on large boxes composed of 2048 ion pairs and another having only 25 ion pairs. The larger boxes are used to obtain coarser (force field-based) information that should be good to reproduce liquid structural motifs at longer distances, while the smaller (still classical) simulation boxes serve the purpose of thoroughly sampling liquid structure to generate initial configurations for multiple 50 ps AIMD runs that when combined produced well-averaged short-distance structural correlations.

**Figure 3:**
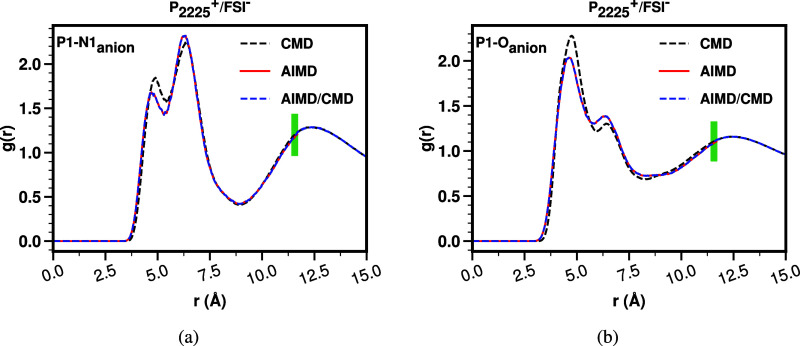
For P_**2225**_^**+**^/FSI^**–**^, an example of radial distribution functions for CMD, AIMD, and the hybrid AIMD/CMD approach. The green vertical bar shows the merging region (Section 2.3.3).

Data from CMD and AIMD trajectories are then combined to get hybrid pair distribution functions, *g*(*r*), examples of which are shown in Figure 3a,b, that accurately capture the conformation of ions at short-range but also the long-range behavior, avoiding the typical sampling and truncation errors of standalone first-principles trajectories and the shortcomings of force fields at shorter distances. An additional advantage is that AIMD trajectories are sampled from constant pressure classical simulations, resulting in each having a different volume consistent with typical fluctuations.

#### 2.3.2 Details of Each Simulation Type

All classical MD simulations were carried out using the GROMACS software package version 4.5.5,^30^ and all AIMD simulations were conducted using the CP2K code version 2023.2 with the built-in QUICK-STEP module.^31^ For each classical box type, ILs were initially packed using the PACKMOL^32^ software and then energy minimized. Three successive equilibration steps were then performed in the NPT ensemble using the V-rescale thermostat^33^ and Berendsen barostat^34^ with 0.2 and 1.0 ps time constants, respectively. The first step was run for 0.2 ns at a pressure of 50 bar and scaled partial charges of 1% of their correct values; the duration of the second step was 2 ns and was run at a pressure of 50 bar and scaled partial charges of 10%, whereas the third step was run for 10 ns (2 ns for the 25 ion pairs) at a pressure of 1 bar and 100% partial charges. After these preliminary equilibration steps at 300 K, we further subjected the systems to an 8 ns simulated annealing scheme in which the temperature was ramped from 300 to 700 K and then brought back to a target temperature of 400 K. Final configurations of these annealing runs were used as initial conditions for 10 ns production runs in the constant pressure and temperature (NPT) ensemble. Annealing and production runs were carried out using the Nosé–Hoover thermostat^35^ and the Parrinello–Rahman barostat^36^ with 0.2 and 1.0 ps time constants, respectively. For our large-box simulations, structures from the last 2 ns were used for data collection. In addition, for comparison to experimental density measurements, we also equilibrated a large box using the same protocol but with a final temperature of 341 K.

For our larger-box simulations, the cutoff for all the nonbonded interactions (van der Waals and electrostatic) was set to 15 Å, and for our smaller-box simulations used as initial conditions for AIMD runs, the cutoff was set at 10 Å. The particle mesh Ewald^37,38^ method was used for the long-range electrostatic interactions with a sixth-order interpolation and Fourier spacing of 0.8 Å. 3D periodic boundary conditions were applied as coded in GROMACS. Equations of motion were integrated using a time step of 1.0 fs with the MD integrator.^39,40^ Classical potential parameters for our simulations, which we provide as GROMACS input files in the Supporting Information, were derived from a combination of sources including OPLS-AA,^41^ the Canongia Lopes and Pádua force fields,^42−45^ Price et al.,^46^ Shimizu et al.,^47^ and Kaminski et al.^48^ Bond, angle, and torsion parameters involving tail oxygen atoms were taken from OPLS-AA,^41^ Weiner et al.,^49^ and Cornell et al.^50^

AIMD calculations were run in the constant volume and temperature (NVT) ensemble at 400 K under periodic boundary conditions using a Nosé–Hoover chain thermostat with a time constant of 1.0 ps. Each of the multiple trajectories was 50 ps in duration with a time step of 1.0 fs. AIMD simulations used the Perdew–Burke–Ernzerhof^51,52^ (PBE) density functional level of theory, the MOLOPT-DZVP-GTH basis set,^53^ the DFT-D3^54^ dispersion correction, and the Goedecker–Teter–Hutter (GTH) pseudopotentials.^55^ A 600 Ry density CUTOFF criterion was used together with REL_CUTOFF set to 60 Ry. 10^–6^ was used as the target accuracy threshold for the self-consistent field (SCF) convergence. The orbital transformation (OT) method^56^ and the FULL_ALL preconditioner were used.

As schematically depicted in Figure 2, ten distinct AIMD simulations were launched for each of the ionic liquids (P_**2225**_^**+**^/FSI^**–**^, P_**222(2O2)**_^**+**^/FSI^**–**^, and P_**222(2S2)**_^**+**^/FSI^**–**^). Initial configurations for these were frames separated by 1 ns from our 10 ns small-box classical production run. This time separation between initial frames guarantees good statistical diversity in starting structures and volumes (densities). In all cases, to adjust the classical potential to the first-principles potential, 50-step geometry optimizations were performed before launching AIMD trajectories. These adjust bond lengths, angles, and dihedral configurations but do not significantly alter the overall liquid structure.

#### 2.3.3 Construction of Hybrid Pair Distribution Functions and *S*(*q*)

In order to compare simulation data with experimental results, we computed three sets of functions, derived from (i) AIMD, (ii) CMD, and (iii) the hybrid approach. For each of the ILs, P_**2225**_^**+**^/FSI^**–**^, P_**222(2O2)**_^**+**^/FSI^**–**^, and P_**222(2S2)**_^**+**^/FSI^**–**^, the AIMD results used the combined data of 10 trajectories merged to become a single file from which *g*(*r*), *S*(*q*), and probability distributions of distances and angles were computed.

Hybrid (AIMD/CMD) *S*(*q*) functions were calculated from hybrid *g*(*r*) functions. Specifically, data points from classical *g*(*r*) functions at distances *r* ≤ 11.56 Å were replaced with the corresponding points from AIMD *g*(*r*) functions. As a result, the hybrid *g*(*r*) consists of AIMD data for *r* ≤ 11.56 Å, and CMD data for *r* > 11.56 Å. Whereas these functions patched well without major discontinuities, we nonetheless used a cubic spline function that ensures up to second derivative smoothness to connect them in a “merging region”. This merging region spans 7 points on either side of *r* = 11.56 Å, with a spacing of 0.02 Å. Within this region, a cubic spline is applied to ensure a smooth transition between the AIMD and classical *g*(*r*) data. Figure 3a,b shows examples of the merged functions.

The computational *S*(*q*) was calculated using equation
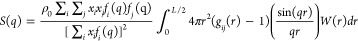
3where *g_ij_*(*r*) is the radial distribution function and *W*(*r*) is a Lorch-type function^57,58^ that minimizes finite box truncation errors. For the classical and AIMD simulations, ρ_o_ is the average total number density over the frames used in each case. For the hybrid method, it is taken as the average number density used in the classical case. As in our prior work, we use the fact that *S*(*q*) can be partitioned into additive ionic and subionic components to derive structural information on different length scales. The definition of subspecies used throughout the manuscript is given by the color-coded fragments in Figure 1.

## 3 Results and Discussion

**Figure 4:**
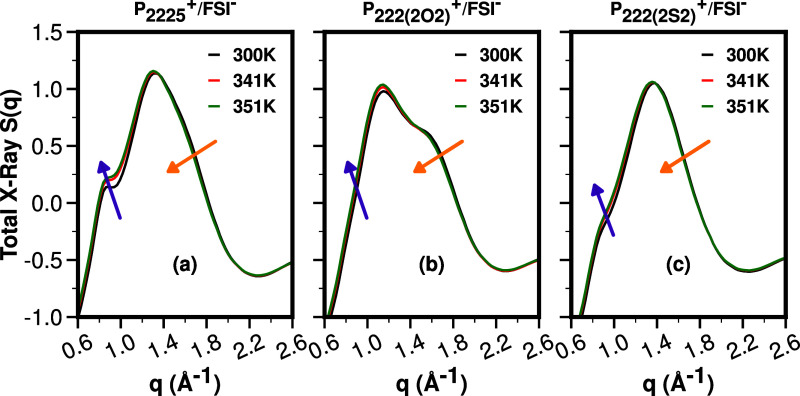
From left to right, the experimental *S*(*q*) for P_**2225**_^**+**^/FSI^**–**^, P_**222(2O2)**_^**+**^/FSI^**–**^, and P_**222(2S2)**_^**+**^/FSI^**–**^, as a function of temperature in the range 300 K through 351 K.

For *q*-values in the 0.6–2.6 Å^–1^ range, associated with adjacency and charge alternation correlations,^14−26^Figure 4 shows for P_**2225**_^**+**^, P_**222(2O2)**_^**+**^, and P_**222(2S2)**_^**+**^ each coupled with FSI^**–**^, the experimental *S*(*q*) in the range 300–351 K; experimental *S*(*q*) functions in a much larger *q*-range are provided in Figure S1. For these ILs, there is no prepeak or first sharp diffraction peak at low *q*, and hence the *q*-region shown in Figure 4 is the most relevant as it encompasses the adjacency (∼1.2–2.2 Å^–1^) and charge alternation (around ∼0.8 Å^–1^) regions.

We see that the temperature dependence of *S* (*q*) in Figure 4a–c is quite weak, and this is particularly the case in the regime we are most interested in studying for this article, associated with the adjacency peak of *S*(*q*) at or above 1.2 Å^–1^ but below 2.2 Å^–1^. Arrows in Figure 4a–c highlight that, in the adjacency region, a very modest shift to lower *q* values occurs with increasing temperature and, for fixed *q* values, a very modest shift to lower intensity. Instead, in the charge alternation region below 1 Å^–1^, the arrow highlights a modest increase in intensity at a fixed *q*.

This very weak dependence of *S*(*q*) on temperature in the adjacency region of *S*(*q*) turns out to be very advantageous computationally, as our simulations are about 50 K higher (400 K) than the highest scattering temperature measured at the APS. Because of the prohibitive cost of first-principles simulations, as a rule of thumb, we prefer to work at single-digit values of the viscosity in units of cP. At room temperature, the viscosities of these ILs are high for our computational purposes, particularly in the cases of P_**2225**_^**+**^/FSI^**–**^ and P_**222(2S2)**_^**+**^/FSI^**–**^; however, Table 1 shows that experimentally predicted viscosities (see the sixth column in Table 1) fall in the desirable single-digit cP range at 400 K.

**Table 1:** Experimentally Measured Physical Properties

Ionic Liquid	a*T*_g_ (K)	Density at 25 °C (298.15 K) (g/mL)	Conductivity (mS/cm)	Viscosity (cP) at 25 °C (298.15 K)	bPredicted Viscosity (cP) 341.15 K (400 K)
P_**2225**_^**+**^/FSI^**–**^	171	1.22	3.5 at 23.1 °C	68	15.8 (4.7)
P_**222(2O2)**_^**+**^/FSI^**–**^	166	1.25	6.0 at 23.4 °C	39	10.8 (3.9)
P_**222(2S2)**_^**+**^/FSI^**–**^	179	1.30	2.6 at 23.4 ^◦^C	87	20.0 (5.9)

a All values are onset temperatures, ± 1 K.

b All data are from this work except (b) where we used the VFT fits from ref (1)

**Figure 5:**
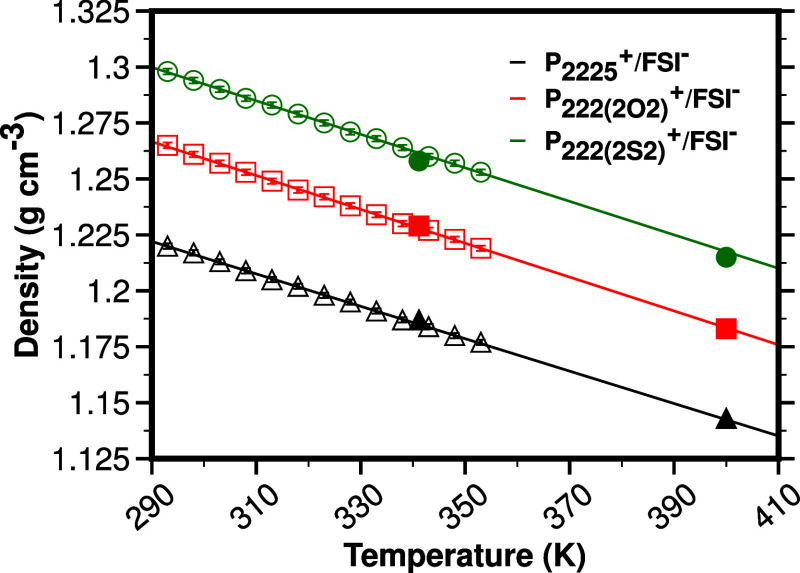
From ref (1), experimental density values as a function of temperature including error bars (green empty circles for P_**222(2S2)**_^**+**^/FSI^**–**^, red empty squares for P_**222(2O2)**_^**+**^/FSI^**–**^, and black empty triangles for P_**2225**_^**+**^/FSI^**–**^) as well as color matching solid-line linear fits to the data.^1^ From the current study, computationally derived densities at 341.15 and 400 K depicted as color-coded solid circles, squares, and triangles. The graph shows that large-box force field-based classical MD results match the experiments densities well (see also Table. S1).

As noted in ref (1) and, as can be gleaned from the values in Table 1, the viscosity of P_**222(2S2)**_^**+**^/FSI^**–**^ is the highest while that of P_**222(2O2)**_^**+**^/FSI^**–**^ is the lowest. If we consider the simulated diffusivity of the different cations as a proxy for the viscosity, we also find the same trend from our classical MD simulations (data not shown). Although it is not surprising that ether substitutions in IL tails lead to reduced viscosity and enhanced mobility,^10−12^ the effect of thioether substitutions on dynamics is less well understood, and we may explore it further in subsequent publications. Figure 5 shows that for each system, the match between classically simulated densities and experiment is excellent, and this can also be gleaned from Table S1. Our focus in this article is on structure, and the comparison of physical properties we have thus far presented provides confidence in the classical models used as an excellent starting point for further in-depth refinement and analysis.

### 3.1 Reciprocal Space Analysis and the Motivation for a Hybrid CMD/AIMD Approach

**Figure 6:**
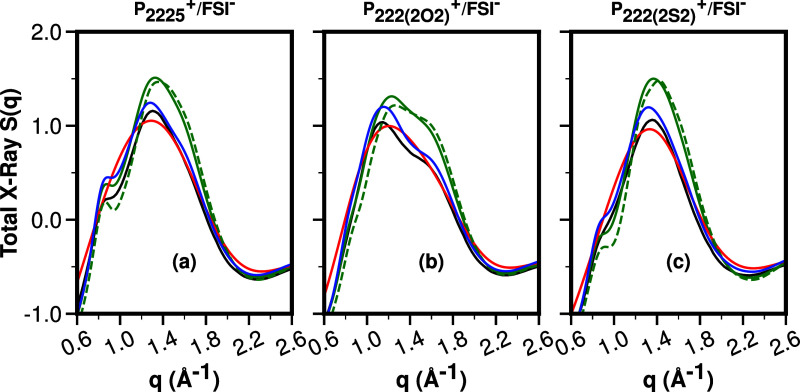
For P_**2225**_^**+**^, P_**222(2O2)**_^**+**^, P_**222(2S2)**_^**+**^, all coupled with FSI^**–**^, the total *S*(*q*) in the *q* regime is between 0.6 and 2.6 Å^–1^ which includes the adjacency and charge alternation regions. Experiment (black line at 351 K), CMD (solid green line at 400 K), CMD (dashed green line at 341.15 K), AIMD (red line at 400 K), and the AIMD/CMD hybrid approach (blue line at 400 K).

Aside from the important discussion on the structural similarities and differences across these novel ILs (Sections 3.2 and 3.3), Figure 6 presents one of the main points our study intends to address. If we focus first on the classical MD results (green solid line at 400 K and dashed line at 341 K), we notice that all features of the experimental *S*(*q*) (black line at 351 K) are captured. In fact, the simulations also capture the very weak temperature dependence of the adjacency peak and the experimental shifts highlighted by the arrows in Figure 4a–c. The literature would accept this level of agreement as very good, but we notice that the intensity of the simulated *S*(*q*) function is obviously larger than the experimental one in the adjacency regime above 1.2 Å^–1^.

On the other hand, it is clear that in the adjacency region the small-box AIMD results that stem from well-time-separated and well-classically-equilibrated initial conditions have improved intensities when contrasted to experiments (red lines compared to black lines in Figure 6). The problem with the first-principles data is that they are essentially featureless, not capturing the different shoulders or subpeaks in the *q*-regime of interest shown in Figure 6.

In other words, whereas the combination of multiple AIMD runs sampled from initial volumes and structures derived from classical MD produces excellent higher q results associated with intramolecular and short-range intermolecular behavior, these are not acceptable at lower *q* values missing the capture of longer range structural features. This is particularly problematic in the charge alternation region at around 0.8 Å^–1^ which is associated with networks. The issue is likely due to system size limitations and not due to poor AIMD sampling, given that we have sampled extensively (10 × 50 ps trajectories in each case), and the viscosity of the three simulated systems is fairly low at 400 K.

We therefore propose an approach in which the short length scale behavior is taken from the first-principles calculations in smaller boxes and the longer range behavior is taken from very large classical simulations. The results are the blue lines in Figure 6a–c where intensity is massively improved with respect to the purely classical studies, and liquid features are recovered when compared with the featureless *S*(*q*) derived from AIMD.

**Figure 7:**
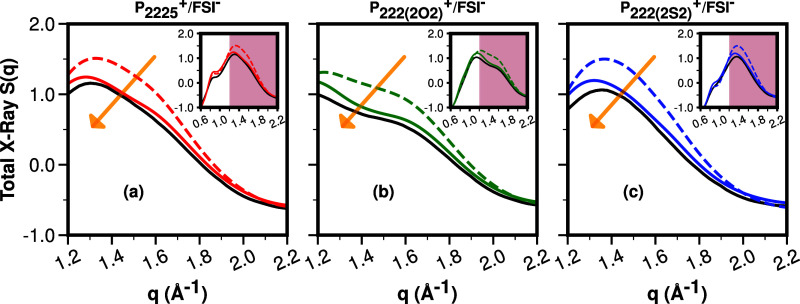
Experimental *S*(*q*) compared to CMD and AIMD/CMD versions of it in a region that focuses on adjacency (intramolecular and nearest neighbor intermolecular) correlations. Experimental (black) and color-coded by IL, CMD (dashed), and AIMD/CMD (solid). The arrow highlights the direction of improvement in the simulated data. Inset shows data on a larger *q*-range.

We are particularly interested in the improvements achieved at shorter distances (adjacency regions), and Figure 7 focuses on those. Specifically, we see that the combined AIMD/CMD approach generates a structure function *S*(*q*) that is very close to that generated from the coherent intensity measured at the APS. In fact, differences in experimental setup such as detector distance or quality of background corrections or choices made in data analysis such as the high *q* cutoffs might result in differences on the order of the deviation between experiment and simulation now seen. We emphasize that this is not your blanket run-of-the-mill “AIMD results are better than CMD results statement” -that is often not true (or at least not tested) in condensed phase simulations-. Instead, for these specific ILs, we prove that the hybrid method truly produces better results in the adjacency region of *S*(*q*), and this is because we are using the short-range AIMD data.

With the new improved structure function in place, we can now analyze the structural features that are primarily responsible for the difference between the CMD and the hybrid CMD/AIMD results and, ultimately, the experiments. This provides an opportunity for us to suggest deficiencies in classical force fields at short range that when mitigated will result in improvements when compared to experiments.

**Figure 8:**
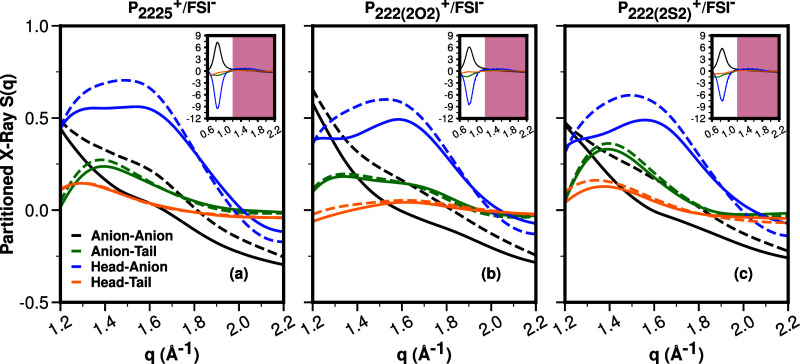
For P_**2225**_^**+**^, P_**222(2O2)**_^**+**^, and P_**222(2S2)**_^**+**^, all coupled with FSI^**–**^. Relevant partial subcomponents of *S*(*q*) from CMD and the hybrid AIMD/CMD approach show significant deviations across methods. Dashed lines are for CMD and solid lines are for the hybrid AIMD/CMD approach. The inset shows results on a larger *q*-range highlighting the charge alternation region with a white background and the adjacency region in pink.

For a deeper dive into the actual structural features that result in such improvements, following prior studies from our group,^14−26^ we partition the total *S*(*q*) into contributions of ionic and subionic species as defined in Figure 1. For *q* values most relevant to adjacency correlations, we plot in Figure 8 the subcomponents that show the largest disparity between the CMD and the hybrid approach. The region plotted is that highlighted in pink in the inset; notice also the massive cancellation of peaks and antipeaks in the charge alternation region (white background portion of the inset) that dwarf the adjacency correlations’ intensities. These massive peaks and antipeaks that cancel are the hallmark behavior for partial subcomponents of *S*(*q*) in all ILs^14−26^ and molten salts;^59−67^ they indicate the existence of charge networks. Even though the subcomponents in the charge alternation region are massively large, because of cancellations, they often only contribute a minor shoulder to the overall *S*(*q*) (see Figures 4 and 6).

Figure 8 shows that significant differences in the adjacency *q*-regime (defined in the plot as between 1.2 and 2.2 Å^–1^) come from anion-related correlations, specifically the anion–anion, cation head–anion, and to a lesser extent the cationic tail–anion correlations (black, blue, and green lines, respectively). Hence, in Section 3.2, we focus on the intramolecular structural behavior of the FSI^**–**^ anion. We will see that differences between CMD and AIMD results for FSI^**–**^ are consistent and are the same across all ILs studied.

### 3.2 A Focus on Intramolecular Features of the FSI^**–**^ Anion

**Figure 9:**
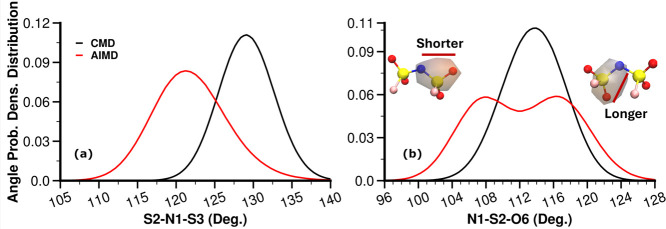
Selected bond angle probability distributions in FSI^**–**^ (see Figure 1 for atomic labels) from the CMD (black line) and AIMD (red line). Corresponding plots for the cases of P_**222(2O2)**_^**+**^/FSI^**–**^ and P_**222(2S2)**_^**+**^/FSI^**–**^ are shown in Figures S2 and S3, respectively.

Figure 8 hints that one should pay close attention to anion structural correlations at a short distance (the adjacency region of *S*(*q*)). Figure 9a but most importantly Figure 9b highlights that there are important differences in real space associated with certain angle distributions in the condensed phase when contrasting classical and quantum simulations. These specific differences are found across all three ILs as can be gleaned from Figures S2 and S3 .

We see from Figure 9b that the AIMD case shows dual probability maxima in the intramolecular anionic N–S–O angle distribution. Because OPLS-type force fields are based on harmonic angle potentials, we believe that these two separate angle configurations cannot be properly captured by CMD. Naturally, classical simulations then give rise to a single maximum somewhere in between the two AIMD peaks. The two configurations do not correspond to two different ionic conformers; instead, they result from the electronic structure of the anion preferring a shorter and a longer intramolecular N–O distance, as depicted in insets associated with Figures 9b, S2b, and S3b.

**Figure 10:**
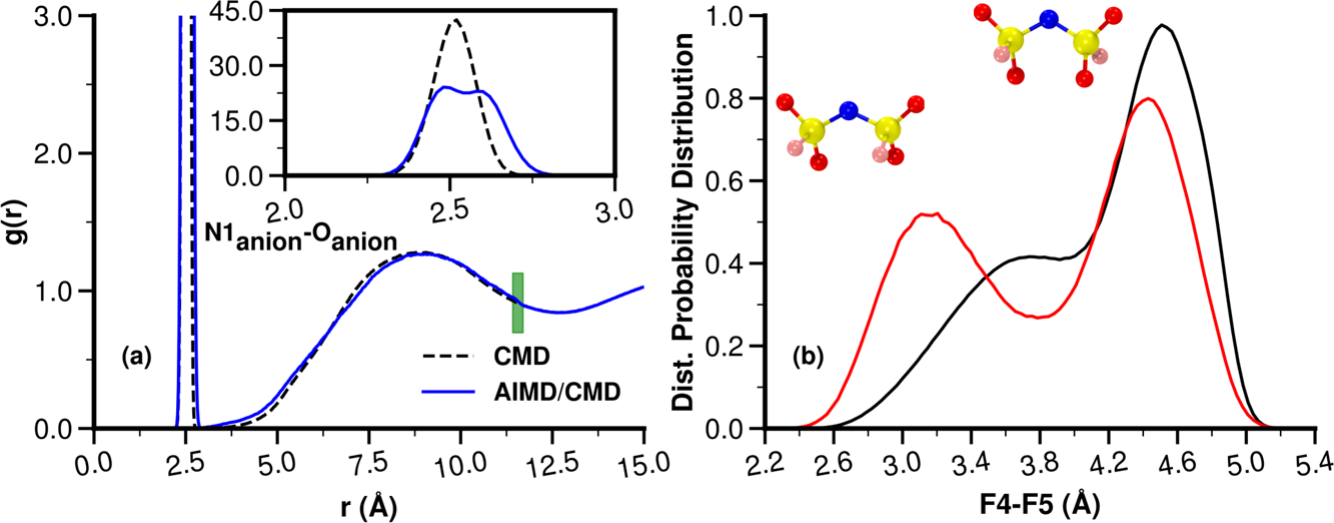
For P_**2225**_^**+**^/FSI^**–**^, (a) example anionic N–O radial distribution function from CMD and the hybrid AIMD/CMD approach. The green vertical bar shows the merging region. The figure is truncated in the *Y* direction for clarity, but the large intramolecular peak highlighting two different intramolecular distances is shown as an inset; this is consistent with the ball and stick anionic representations in the inset of Figure 9b. (b) Anionic intramolecular F–F distance distribution showing differences in cisoid and transoid conformer probabilities (red line is for AIMD and black line for CMD) across methods. Corresponding figures showing consistently similar findings for the behavior of FSI^**–**^ in the cases of P_**222(2O2)**_^**+**^/FSI^**–**^ and P_**222(2S2)**_^**+**^/FSI^**–**^ are shown in Figures S4 and S5, respectively.

Notice that concomitant with this dual peak in the angle distribution, there is also a double peak in the intramolecular portion of *g*(*r*) for the N–O pair, as can be gleaned from the inset in Figure 10a; this makes the intramolecular *g*(*r*) peak broader in the AIMD (or hybrid AIMD/CMD) case. Figure 9a also shows that the most probable S–N–S angle in the CMD case is about 10° larger, and the probability distribution is narrower when compared to AIMD.

When considering the F atoms in the FSI^**–**^ ion, transoid and cisoid conformations are expected. Figure 10b shows that these are captured by the classical and AIMD simulation schemes but at different F–F distances; whereas the transoid state is preferred by both simulation schemes, this is more prominent in the CMD study. Seen as a whole, it would appear that improvements in the way FSI^**–**^ is commonly treated should result in more accurate simulations that better reproduce *S*(*q*). However, changes may require not just an adjustment of force field parameters but also likely a change in the functional form of the potential.

### 3.3 Cation and Cation–Anion Correlations at Short Range

Because of the many rotatable bonds in P_**2225**_^**+**^, P_**222(2O2)**_^**+**^, and P_**222(2S2)**_^**+**^, it is expected that multiple conformers will exist for these ions in solution. Whereas Section 3.2 highlights commonalities in the behavior of the FSI^**–**^ anion across ILs (and differences across methods), Section 3.3 showcases significant differences in the intramolecular cationic behavior across ILs (and across methods).

**Figure 11:**
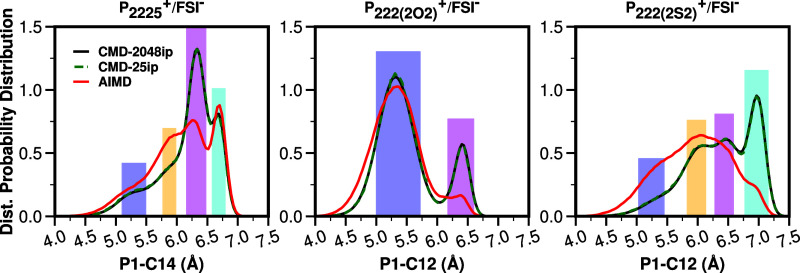
For P_**2225**_^**+**^/FSI^**–**^, P_**222(2O2)**_^**+**^/FSI^**–**^, and P_**222(2S2)**_^**+**^/FSI^**–**^, cationic intramolecular P1-CT distance distribution highlights differences and similarities across methodologies. P1 is the phosphorus atom and CT is the terminal carbon atom of the longest side chain.

First, we would like to dispel the idea that intramolecular conformational differences across methods may come from the effect of box size (25 ion pairs vs 2048 ion pairs) instead of the methods. For P_**2225**_^**+**^/FSI^**–**^, P_**222(2O2)**_^**+**^/FSI^**–**^, and P_**222(2S2)**_^**+**^/FSI^**–**^, Figure 11 shows the distance distribution between cationic P and the terminal C atom (CT) in the longest cationic tail. We see that the results for classical boxes of 2048 and 25 ion pairs are for all practical purposes, indistinguishable. On the contrary, first-principles sampling of these, which uses as initial conditions well-equilibrated and well-time-separated configurations derived from small classical MD boxes, results in significantly different probability distributions. Our AIMD calculations are also very well sampled, given that for each system we run 10 × 50 ps MD segments and the viscosity is low.

We learn from Figure 11 that CMD and AIMD produce conformers at about the same P-CT distance value (highlighted with vertical color boxes); yet, the probability of each conformer is different across methods. Interestingly, independent of the method, P_**222(2O2)**_^**+**^ always appears to show only two conformers (the most abundant is the one with the curled version of the tail), whereas P_**2225**_^**+**^ and P_**222(2S2)**_^**+**^ appear to show at least four conformers along this particular order parameter. The most notable difference across methods is for P_**222(2S2)**_^**+**^/FSI^**–**^, where CMD prefers a more extended version of the longer tail, whereas AIMD prefers a more curled version of it. Notice that whereas both P_**222(2O2)**_^**+**^ and P_**222(2S2)**_^**+**^ cations prefer curled tails, the nature of curling is different.

**Figure 12:**
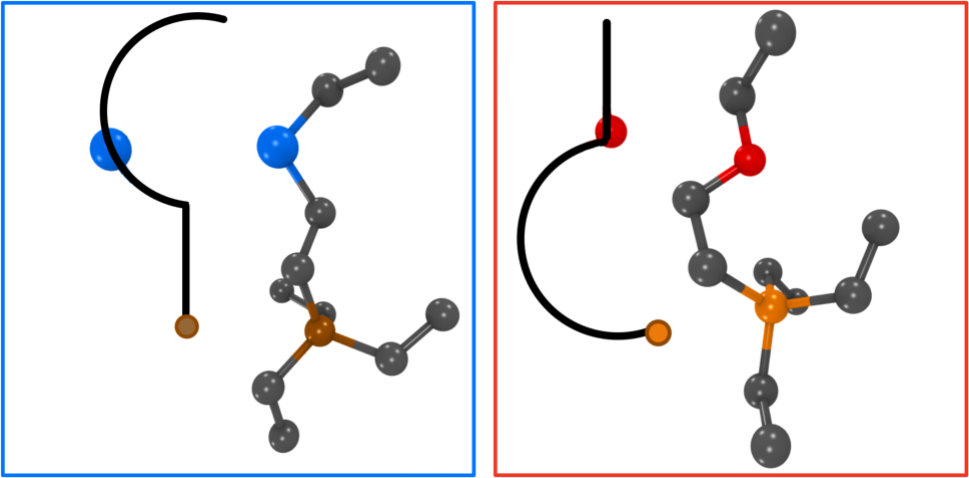
For P_**222(2S2)**_^**+**^ (left) and P_**222(2O2)**_^**+**^ (right), and derived from AIMD simulations, the most likely longest tail conformation. Hooks are cartoons representing the longest tail and the corresponding positions of P and S, or P and O.

Derived from AIMD runs, Figure 12 shows the most typical cationic conformer in the case of P_**222(2S2)**_^**+**^/FSI^**–**^ and P_**222(2O2)**_^**+**^/FSI^**–**^, where the distance between P and CT is about 6 and 5.3 Å, respectively. For P_**222(2S2)**_^**+**^ and P_**222(2O2)**_**^+^,** these correspond to ionic structures in the orange and blue boxes in Figure 11, respectively. Figure 12 is meant to emphasize that, in the most abundant cationic conformation, tails curl differently in the case of the substitution of the O and S, and we highlight this pictorially with hooks. Each hook represents the longest cationic tail (including the P atom) and shows the relative position of S and P, or O and P. In the case of the S substitution, the most likely conformer has curling that is most notable toward the end of the tail but in the case of O it is toward the beginning of the tail. Furthermore, O appears to be at the end of the curl but S is in the middle of it.

#### 3.3.1 How Do These Short-Range Conformational Differences Across Methods Affect the Pair Distribution Function?

**Figure 13:**
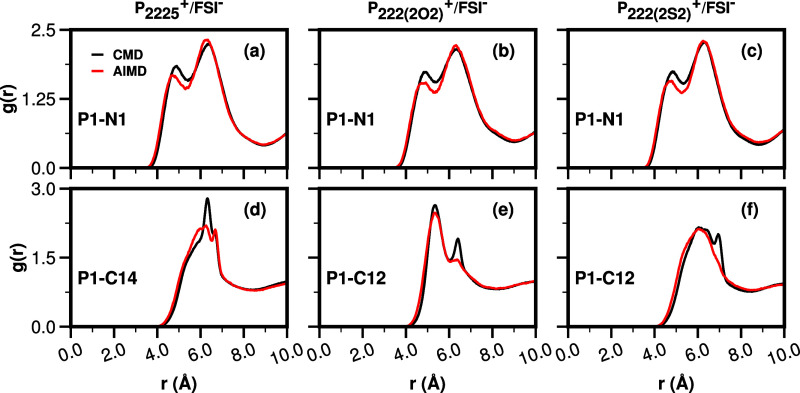
For P_**2225**_^**+**^, P_**222(2O2)**_^**+**^, and P_**222(2S2)**_^**+**^, all coupled with FSI^**–**^, (top panel) cation–anion P–N pair distribution function; (bottom panel) cationic P-CT pair distribution functions, comparing the effect of simulation type.

Because changes in the intramolecular conformation of the anion are essentially the same across ILs when comparing CMD and AIMD, and because it is likely that differences in the long cationic tail conformation do not affect the close contact between P and FSI^**–**^, one could intuitively predict that *g*(*r*) should see similar differences across methods for the three FSI^**–**^-based ILs. This is exactly what the top panel in Figure 13 is showing. Specifically, the first peak is lower in the AIMD case for all three FSI^**–**^-based ILs and the second peak is moderately higher, particularly in the case of P_**2225**_^**+**^.

The opposite is true in the case of conformations purely associated with cations for which each IL shows changes that are unique, as can be gleaned from the bottom panel of Figure 13. When we consider cation–cation correlations, we see that the classical MD has sharper features at short-range whereas AIMD smooths those out, and this is completely consistent with the distance distributions in Figure 11 where we also find that CMD has sharper peaks.

## 4 Conclusions

This article explores the family of FSI^**–**^-based ILs coupled with the P_**2225**_^**+**^, P_**222(2O2)**_^**+**^, and P_**222(2S2)**_^**+**^ cations. There are multiple interesting findings, for example, the single atom substitution causes significant cationic conformational changes, and these are reflected in the structure function as well as other properties, most notably the viscosity. Both P_**222(2O2)**_^**+**^ and P_**222(2S2)**_^**+**^ display curled tail conformations that are statistically favorable; however, curling looks quite different in each case. This work develops a methodology that improves the computational *S*(*q*), but most importantly, it allows us to test whether AIMD ionic structures in the condensed phase improve over force field-based results. For a given first-principles method, such a comparison is neither trivial, nor obvious for an arbitrary system; quite the opposite. Such a comparison is not straightforward to make, given that one cannot produce a proper *S*(*q*) for ILs on small AIMD simulation boxes alone. This is because *S*(*q*) needs to capture the existence of long percolating networks. By merging short-range results from AIMD with the longer range data from classical force field simulations, we can observe an improvement in *S*(*q*) particularly in the adjacency *q*-regime. This implies that the intraionic conformations and the correlations between adjacent ions derived from condensed phase AIMD are superior in this specific instance, thus providing a handle on specific force field features that may need improvement. As such a finding is obtained in the condensed phase, it is significantly more valuable than improving gas-phase forces and energetics. One curious finding is dual minima along anionic angle coordinates that is likely the cause for a double intramolecular peak in *g*(*r*) for the anion. Such double minima may require not just different force field parameters but a different functional form of the angle potential.

## Supporting Information Available

Table with simulated temperatures, densities, and predicted experimental densities, experimental *S*(*q*) on a large *q*-range, selected angle probability distributions, selected pair distribution function and anionic intramolecular F–F distance distributions (PDF)
Topology files that can be used with the software GROMACS (TXT)
Topology files that can be used with the software GROMACS (TXT)
Topology files that can be used with the software GROMACS (TXT)
