# Supplementary material for: Structure of Novel Phosphonium-Based Ionic Liquids with S and O Substitutions from Experiments and a Mixed Quantum-Classical Approach
Source: J Phys Chem B. 2025 Mar 27;129(14):3691–701. doi: 10.1021/acs.jpcb.5c00129 (PMC11995385; doi:10.1021/acs.jpcb.5c00129)
Supplement: Supplementary file 1 — jp5c00129_si_001.pdf [file jp5c00129_si_001.pdf]

Supporting Information: Structure of Novel Phosphonium-Based Ionic Liquids with S and O Substitutions from Experiments and a Mixed Quantum-Classical Approach

Raphael Ogbodo,[†] Gobin Raj Acharya,[‡] Ho Martin Yuen,[¶] Nicole Zmich,[¶] Furong Wang,[§] Hideaki Shirota,[‖] Sharon I. Lall-Ramnarine,[*,¶] James F. Wishart,[*,§] Andrew J. Nieuwkoop,[*,‡] and Claudio J. Margulis[*,†]

†*Department of Chemistry, The University of Iowa, Iowa City, IA 52242, United States*

‡*Department of Chemistry and Chemical Biology, Rutgers University, Piscataway, New Jersey 08854, United States*

¶*Department of Chemistry, Queensborough Community College-CUNY, Bayside, New York 11364, United States*

§*Chemistry Division, Brookhaven National Laboratory, Upton, New York 11973-5000, United States*

‖*Department of Chemistry, Chiba University, Chiba 263-8522, Japan*

E-mail: slallramnarine@qcc.cuny.edu; wishart@bnl.gov; an567@chem.rutgers.edu; claudio-margulis@uiowa.edu

Contents

S.1 Additional Tables and Figures S3

References S6

S.1 Additional Tables and Figures

Table S.1: Ionic liquid densities at T=$\langle T_{NPT} \rangle$ from classical MD simulations compared with experimental fits to equation $\rho(T) = c_\rho T + \rho_o$ from reference S1.

Ionic Liquid	$\langle T_{NPT} \rangle$ (K)	Density (g cm^{-3})	
		Simulation	Expt. fit
P_{2225}^+/FSI$^-$	400 (341.15)	1.14 (1.19)	1.14 (1.19)
$P_{222(2O2)}^+$/FSI$^-$	400 (341.15)	1.18 (1.23)	1.18 (1.23)
$P_{222(2S2)}^+$/FSI$^-$	400 (341.15)	1.22 (1.26)	1.22 (1.26)

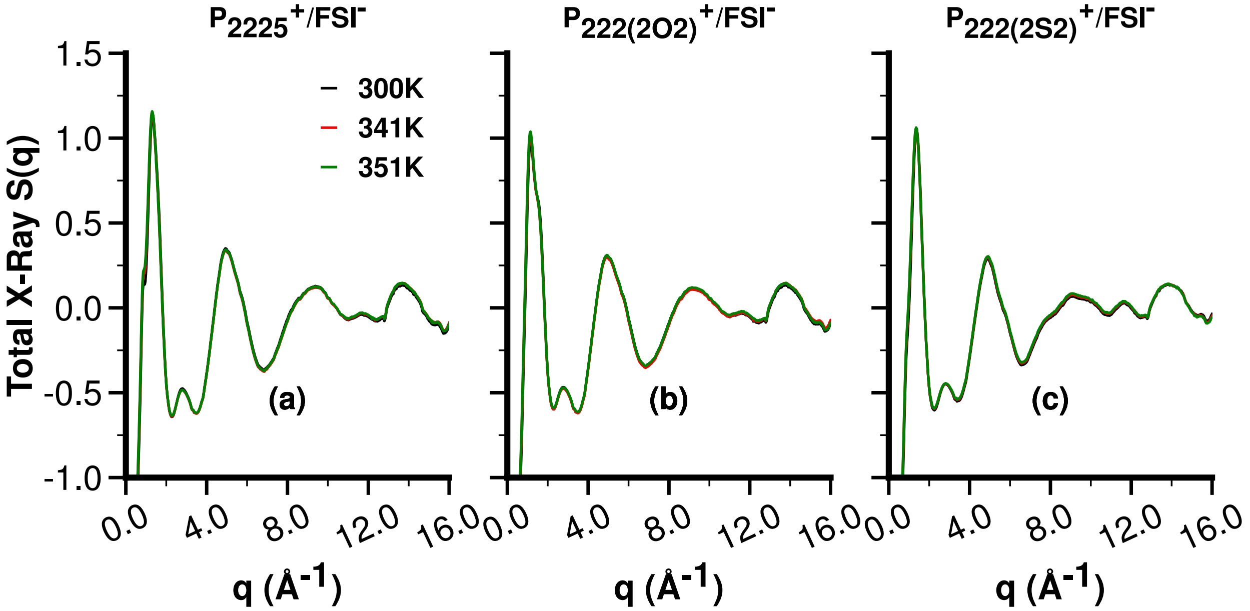

Figure S1: From left to right, the experimental S(q) for P_{2225}^+/FSI$^-$, $P_{222(2O2)}^+$/FSI$^-$, and $P_{222(2S2)}^+$/FSI$^-$, at three different temperatures; see Fig. 4 instead for the smaller q-range associated with adjacency and charge alternation correlations. As can be gleaned from the figure, the temperature dependence of S(q) is quite weak.

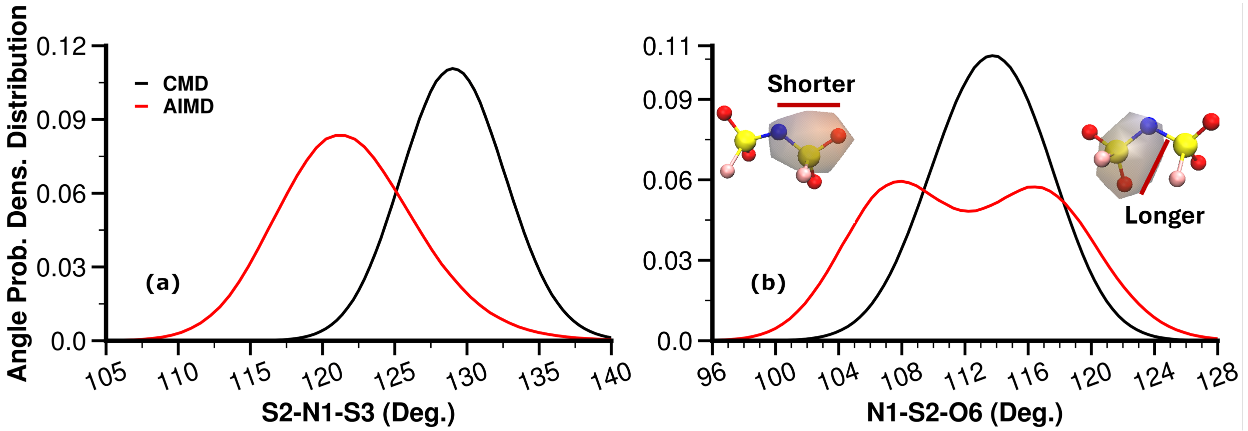

Figure S2: For $P_{222(2O2)}^+$/FSI$^-$, selected bond angle probability distributions in FSI$^-$ (see Fig. 1 for atomic labels) from CMD (black line) and AIMD (red line). Corresponding plots for P_{2225}^+/FSI$^-$ and $P_{222(2S2)}^+$/FSI$^-$ are shown in Figs. 9 and S3 respectively.

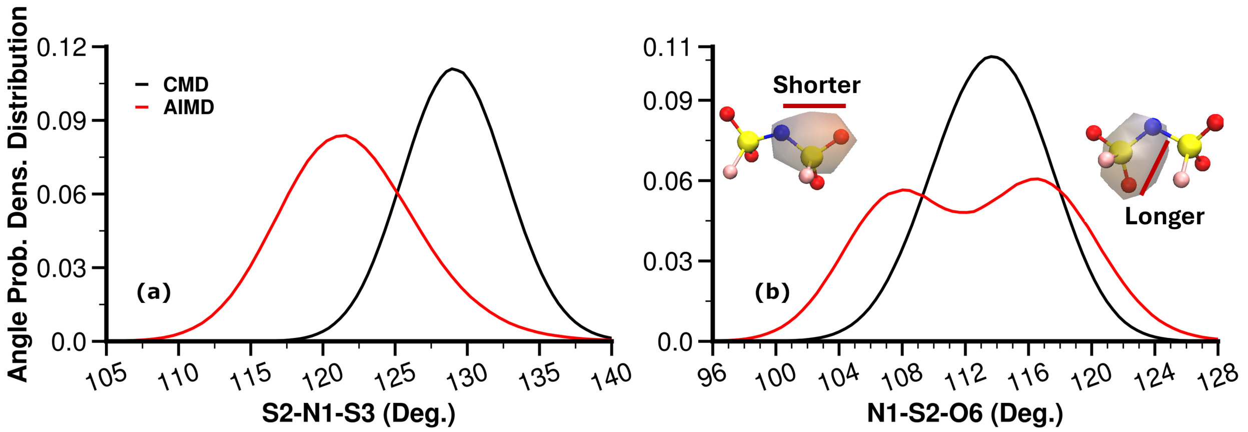

Figure S3: For $P_{222(2S2)}^+$/FSI$^-$, selected bond angle probability distributions in FSI$^-$ (see Fig. 1 for atomic labels) from CMD (black line) and AIMD (red line). Corresponding plots for P_{2225}^+/FSI$^-$ and $P_{222(2O2)}^+$/FSI$^-$ are shown in Figs. 9 and S2 respectively.

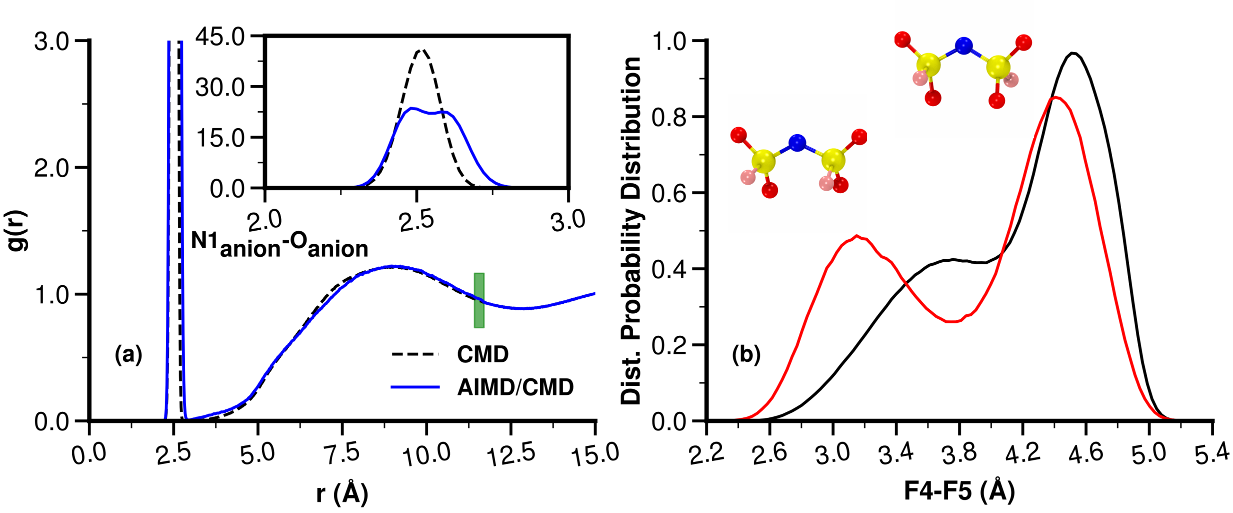

Figure S4: For $P_{222(2O2)}^{+}$/FSI^{-}, (a) example anionic N-O radial distribution function from CMD and the hybrid AIMD/CMD approach. The green vertical bar shows the merging region. The figure is truncated in the Y direction for clarity, but the large intramolecular peak highlighting two different intramolecular distances is shown as an inset. (b) Anionic intramolecular F-F distance distribution showing differences in cisoid and transoid conformer probabilities (red line is for AIMD and black line for CMD) across methods. Notice how similar these plots are to those for P_{2225}^{+}/FSI^{-} in Fig. 10 and for $P_{222(2S2)}^{+}$/FSI^{-} in Fig. S5.

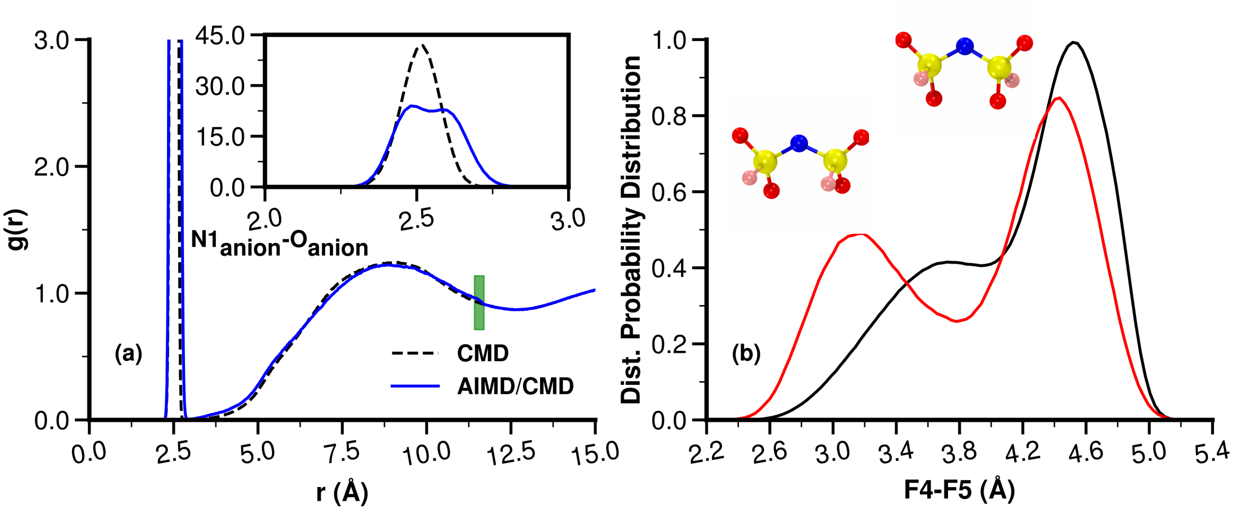

Figure S5: For $P_{222(2S2)}^{+}$/FSI^{-}, (a) example anionic N-O radial distribution function from CMD and the hybrid AIMD/CMD approach. The green vertical bar shows the merging region. The figure is truncated in the Y direction for clarity, but the large intramolecular peak highlighting two different intramolecular distances is shown as an inset. (b) Anionic intramolecular F-F distance distribution showing differences in cisoid and transoid conformer probabilities (red line is for AIMD and black line for CMD) across methods. Notice how similar these plots are to those for P_{2225}^{+}/FSI^{-} in Fig. 10 and for $P_{222(2O2)}^{+}$/FSI^{-} in Fig. S4.

References

(S1) Ando, M.; Ohta, K.; Ishida, T.; Koido, R.; Shirota, H. Physical Properties and Low-Frequency Polarizability Anisotropy and Dipole Responses of Phosphonium Bis(fluorosulfonyl)amide Ionic Liquids with Pentyl, Ethoxyethyl, or 2-(Ethylthio)ethyl Group. *J. Phys. Chem. B* **2023**, *127*, 542–556.
